# Terahertz microscopy using laser feedback interferometry based on a generalised phase-stepping algorithm

**DOI:** 10.1038/s41598-024-53448-8

**Published:** 2024-02-08

**Authors:** Daniel Mohun, Nikollao Sulollari, Mohammed Salih, Lianhe H. Li, John E. Cunningham, Edmund H. Linfield, A. Giles Davies, Paul Dean

**Affiliations:** School of Electronic and Electrical Engineering, University of Leeds, Leeds, LS2 9JT UK

**Keywords:** Applied optics, Lasers, LEDs and light sources, Optical techniques

## Abstract

In this paper we report an improved method of coherent sensing through the use of a generalized phase-stepping algorithm to extract magnitude and phase information from interferometric fringes acquired by laser feedback interferometry (LFI). Our approach allows for significantly reduced optical sampling and acquisition times whilst also avoiding the need for fitting to complex models of lasers under optical feedback in post-processing. We investigate theoretically the applicability of this method under different levels of optical feedback, different laser parameters, and for different sampling conditions. We furthermore validate its use experimentally for LFI-based sensing using a terahertz (THz)-frequency laser in both far-field and near-field sensing configurations. Finally we demonstrate our approach for two-dimensional nanoscale imaging of the out-of-plane field supported by individual micro-resonators at THz frequencies. Our results show that fully coherent sensing can be achieved reliably with as little as 4 sampling points per imaging pixel, opening up opportunities for fast coherent sensing not only at THz frequencies but across the visible and infra-red spectrum.

## Introduction

Laser feedback interferometry (LFI)^1^ is a powerful and versatile sensing technique in which radiation emitted from a laser interacts with an external target and is subsequently reinjected into the laser cavity. The optical mixing [‘self-mixing’ (SM)] that occurs between the intra-cavity and reinjected fields causes, under certain conditions, predictable and controllable perturbations to the laser operation that depend on both the magnitude and phase of the reinjected field^2,3^. In this way the optical properties of the external target may be transferred, through the SM effect, to measurable changes in laser operating parameters.

The experimentally simple form of the LFI scheme, combined with its coherent sensing capability, has motivated its use within a variety of class-A and class-B laser systems and across a wide range of applications spanning visible, infrared and terahertz (THz) regions of the spectrum. Such applications have included metrology^4^, coherent imaging^5–7^, materials analysis^8,9^, gas sensing and spectroscopy^10,11^, Doppler flow measurements^12^, three-dimensional imaging^13,14^, vibrometry^15^, and displacement sensing^16,17^. Furthermore, the SM effect has more generally been exploited for the measurement of fundamental laser parameters including the emission spectrum^18^, laser linewidth^19,20^ and linewidth enhancement factor^21,22^.

One particularly notable application of LFI that has attracted significant interest of late has been scattering-type scanning near-field optical microscopy (s-SNOM), a powerful imaging technique that allows for probing of nanostructures and nanomaterials with resolutions far beyond the diffraction limit^23^. This is achieved by confinement of radiation to a small scanning probe, whereby light-matter interactions occurring in the near-field of the probe can be sensed via dynamic perturbations to the probe scattering efficiency. Notably the high sensitivity of LFI has been exploited recently, in conjunction with the high output powers and low phase-noise afforded by THz-frequency quantum cascade laser (QCL) sources, to enable THz-s-SNOM operating at frequencies beyond 2 THz. This has opened up new opportunities for THz measurements on the micro- and nano-scale including the mapping of charge carriers in semiconductors and nanostructures^24–26^, investigation of plasmon and phonon polaritons in emerging two-dimensional materials^27–29^, and the microscopic investigation of metamaterials and micro- and nano-scale resonators^30–32^.

Despite these successes, however, one limitation of LFI in both far- and near-field implementations stems from the need to acquire full interferometric signals for reliable extraction of amplitude and phase information from a target. This is most commonly achieved by mechanical extension of the optical beam path^13,33^, which results in slow data acquisition. Alternatively, interferometric fringes can be acquired by fast electronic modulation of the laser emission frequency^14,34^. However, modulation rates may then be restricted by the available sampling and signal processing bandwidth^35^. These issues are compounded further by the challenge of detecting a typically small SM voltage perturbation superimposed on a large quiescent signal, which demands long integration times to achieve high signal-to-noise ratios. This is particularly applicable in THz-s-SNOM in which the scattering efficiently of the nanometric probe scales approximately as ~ λ^−4^ and is therefore extremely low at THz frequencies. One means to address these challenges that has been implemented for phase-shifting interferometry at visible and near-infrared wavelengths^36^, enabling applications including digital holography^37,38^ and optical coherence tomography^39,40^, is through reduced optical sampling of the interferometric signals. However the adoption of this approach in LFI is non-trivial owing to the non-sinusoidal nature of the interferometric signals, which are dependent on the strength of optical feedback as well as operating parameters of the laser. This has been addressed recently using a theoretical model encompassing a first-order expansion of the laser frequency under feedback^41^, whereby it has been found that amplitude and phase information can be extracted with significantly reduced optical sampling.

In this paper we report an improved method of coherent sensing through the use of a generalised phase-stepping algorithm (GPSA) to extract magnitude and phase information from interferometric fringes acquired by LFI. Our approach, adopted from phase-shifting interferometry^36^, allows for significantly reduced optical sampling and acquisition times whilst also avoiding the need for fitting to complex models of lasers under optical feedback in post-processing. We investigate theoretically the applicability of this method under different levels of optical feedback, different laser parameters, and for different sampling conditions. We furthermore validate its use experimentally for LFI-based THz sensing in both far-field and near-field sensing configurations. Finally we demonstrate our approach for two-dimensional nanoscale imaging of the out-of-plane field supported by individual micro-resonators at THz frequencies. Our results show that fully coherent sensing can be achieved reliably with as little as 4 sampling points per imaging pixel, opening up opportunities for fast coherent sensing not only at THz frequencies but across the visible and infra-red spectrum.

## Results

### Theoretical evaluation of generalised phase stepping algorithm for LFI

In the standard arrangement of LFI a fraction of radiation emitted from a laser is reinjected into the laser cavity after reflection or scattering from an external target. The reinjected radiation interferes with the field in the laser cavity, causing a change in carrier density, via the self-mixing effect, that depends not only on the magnitude but also the phase of the reinjected field^2,3^. In turn this perturbation to the carrier density induces variations in the optical power, lasing frequency and, in the case of semiconductor lasers, the laser terminal voltage. As first presented in the seminal work of Lang and Kobayashi (L–K)^42^, for small perturbations in carrier density this voltage perturbation (referred to herein as the ‘SM voltage’) can be described (see Methods) according to the relationship

1 $$ V_{{{\text{SM}}}} = \beta \cos \left( {\phi_{L} - \phi } \right). $$

Here $\phi_{L} = \frac{{4\pi L_{{{\text{ext}}}} \nu }}{c}$ is the round-trip phase accumulation in the external cavity of length $L_{{{\text{ext}}}}$ formed between laser facet and target, $\nu$ is the lasing frequency under feedback and $\phi$ is the phase response of the target. The proportionality factor $\beta$ is proportional to the fraction of emitted radiation that is coupled coherently to the laser mode after reflection or scattering from the target after accounting for loss due to attenuation in the external cavity, spatial mode mismatch between the reflected and the cavity mode, and other optical losses. Moreover, $\beta$ can be linked directly to the dimensionless feedback parameter $C$ that defines the strength of optical feedback^1,3^.

As can be seen from Eq. 1, variation of the round-trip phase arising through either mechanical extension of the external cavity or electronic control of the laser frequency induces a series of interferometric fringes in the demodulated SM voltage signal. The complex magnitude of these fringes is directly proportional to the complex reinjected field in the regime of weak feedback. Together, $\beta$ and $\phi$ thereby characterise the optical response of the target from which its complex permittivity^8,34^ or, in the case of s-SNOM, the complex scattering efficiency of the modulated s-SNOM probe in the near-field of the sample can be inferred^27,32,33,43^.

As described by the L–K formalism, the modification to the laser carrier population that is responsible for the SM voltage signal also induces a perturbation to the laser frequency, $\nu$. This effect is encapsulated through the transcendental *excess phase equation* (Eq. 6 in Methods), which relates the round-trip phase under feedback $\phi_{L}$ to the phase $\phi_{L,s} = \frac{{4\pi L_{{{\text{ext}}}} \nu_{s} }}{c}$ calculated for the unperturbed frequency of the solitary laser, $\nu_{s}$. As a result the shape and form of the interferometric fringes described by Eq. 1 are inherently dependent on the strength of optical feedback, quantified by the feedback parameter $C$, as well as the linewidth enhancement factor of the laser, $\alpha$. Nevertheless, in the limit of weak feedback ($C <$1) the perturbed laser frequency is approximately equal to that of the solitary laser, $\nu \approx \nu_{s}$, such that $\phi_{L} \approx \phi_{L,s}$. In this case the SM voltage signal closely follows a cosinusoidal dependence on $\phi_{L,s}$, i.e. $V_{{{\text{SM}}}} \approx \beta \cos \left( {\phi_{L,s} - \phi } \right)$. Crucially, under these conditions, the interferometric fringes encoded within the SM voltage can be reduced to a close approximation by a series of discrete voltage measurements $V_{{{\text{SM}},i}}$, where $i = 0 \to \left( {N - 1} \right)$, taken at $N >$ 3 arbitrary but known phase points $\phi_{L,s} = \phi_{i}$ equally spaced over a single interferometric fringe. Estimates of the true magnitude $\beta$ and phase $\phi$ may then be estimated from the $N$ voltage measurements by applying a generalised phase-stepping algorithm (GPSA). The algorithm used here^36^ models the self-mixing voltage according to the relationship

2 $$ {\text{V}}_{{{\text{SM}},i}} = a_{0} + \beta_{m} {\text{cos}}\left( {\phi_{i} - \phi } \right) = { }a_{0} + { }a_{1} \cos \phi_{i} + { }a_{2} \sin \phi_{i} , $$

in which $a_{0}$ is a constant voltage offset, $a_{1} = \beta_{m} \cos \phi_{m}$ and $a_{2} = \beta_{m} \sin \phi_{m}$, with $\beta_{m}$ and $\phi_{m}$ denoting the output parameters of the algorithm. To solve for the parameters $a_{0}$, $a_{1}$ and $a_{2}$ we apply a least-squares regression (see Methods). Finally the magnitude $\beta_{m}$ and phase $\phi_{m}$ of the SM voltage signal can be obtained from the relations

3 $$ \beta_{m} = \sqrt {a_{1}^{2} + a_{2}^{2} } $$

and

4 $$ \phi_{m} = \tan^{ - 1} \frac{{a_{2} }}{{a_{1} }}. $$

As will be shown, the accuracy of ${\beta }_{m}$ and ${\phi }_{m}$ extracted through this approach depends not only on the chosen value of $N$ but also the phase response of the target itself, as well as the feedback parameter $C$ and linewidth enhancement factor $\alpha$, both of which influence the shape of the interferometric fringes encoded in the laser voltage.

**Figure 1:**
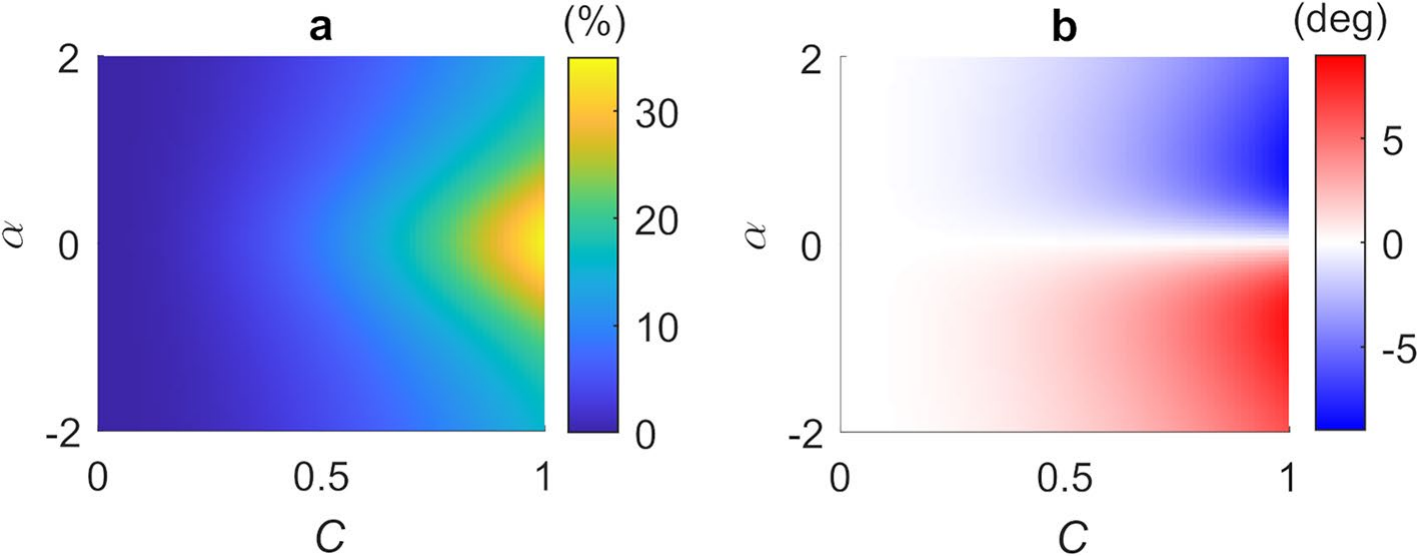
(**a**) Percentage error in the extracted amplitude and (**b**) absolute error in the extracted phase of LFI fringes, extracted using the GDRA in the limit ${\varvec{N}} \to \infty$, shown as a function of feedback parameter ${\varvec{C}}$ and linewidth enhancement factor of the laser ${\varvec{\alpha}}$.

Figure 1a illustrates the percentage error in the fringe magnitude extracted using the GPSA, $e_{A} = \left( {\frac{{\beta_{m} - \beta }}{\beta }} \right) \times 100$, in the limit $N \to \infty$ when applied to a numerically synthesised SM voltage signal described by Eq. 1 (see Methods), for varying combinations of $C$ and $\alpha$. Figure 1b similarly shows the absolute error in determination of the target phase,$e_{\phi } = \phi_{m} - \phi$. In this limit of large $N$ both $e_{A}$ and $e_{\phi }$ depend solely on the parameters $C$ and $\alpha$; these error values represent the fundamental limits of the GPSA approach imposed by the deviation of $V_{{{\text{SM}}}}$ from a purely cosinusoidal function. As expected, for extremely weak feedback ($C <$ 0.1), for which $V_{{{\text{SM}}}}$ closely approximates a cosinusoidal dependence on $\phi_{L,s}$ (see Supplementary Information Figure S1(a)), the errors are small with $e_{A} <$ 0.4% and $e_{\phi } <$ 0.08°. Even with $C =$ 0.5, which is typical for many LFI systems employing THz QCLs, $e_{A}$ remains below 10% and $e_{\phi }$ below 2° according to Fig. 1 (see also Figure S1(b)), which may be considered acceptable for many applications. Indeed, the phase noise associated with frequency instability due to thermal drift of the laser source can often exceed this value^13,14^. For stronger feedback with 0.5$< C <$1, however, the GPSA performs poorly with $e_{A}$ exceeding 30% in cases although with $e_{\phi }$ still remaining below 9°.

**Figure 2:**
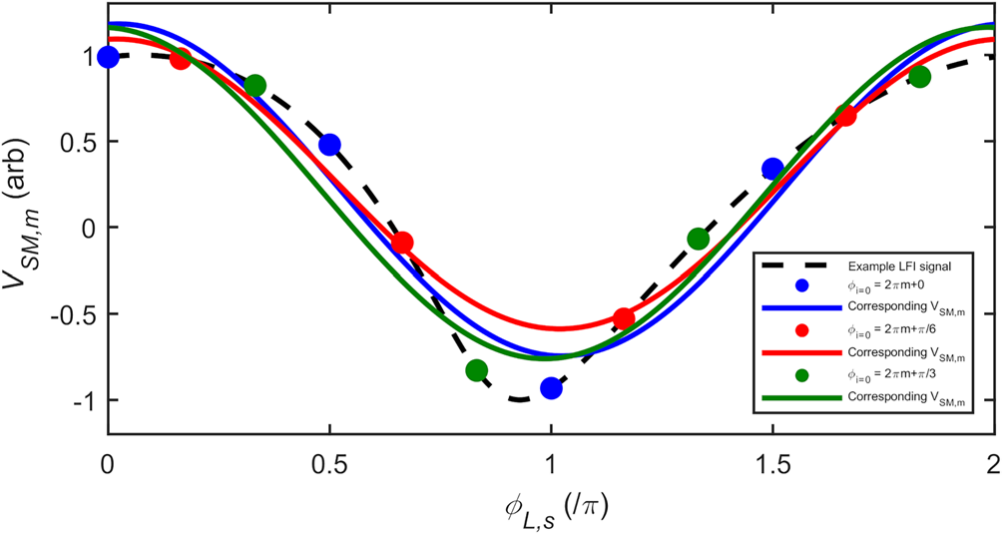
Synthesised LFI signal with ${\varvec{C}} =$ 0.5, ${\varvec{\alpha}} =$ 0.5, amplitude ${\varvec{\beta}} =$1 and phase $\phi =$0 (black dashed line) along with the SM voltages ${\varvec{V}}_{{{\mathbf{SM}},{\varvec{i}}}}$ sampled in three exemplar sets of ${\varvec{N}} = 4$ equally spaced phase points $\phi_{{{\varvec{L}},{\varvec{s}}}} = \phi_{{\varvec{i}}}$ (with ${\varvec{i}} = 0 \to 3)$ with $\phi_{{{\varvec{i}} = 0}} = 2\user2{\pi m} + 0$ (blue circles), $2\user2{\pi m} + {\varvec{\pi}}/6$ (red circles) and $2\user2{\pi m} + {\varvec{\pi}}/3$ (green circles). Also shown (coloured solid lines) are the corresponding functions ${\varvec{V}}_{{{\mathbf{SM}},{\mathbf{m}}}} = {\varvec{\beta}}_{{\varvec{m}}} \cos \left( {\phi_{{{\varvec{L}},{\varvec{s}}}} - \phi_{{\varvec{m}}} } \right)$ plotted using the values of ${\varvec{\beta}}_{{\varvec{m}}}$ and $\phi_{{\varvec{m}}}$ determined from the GDRA applied to each set of $\phi_{{\varvec{i}}}$.

**Figure 3:**
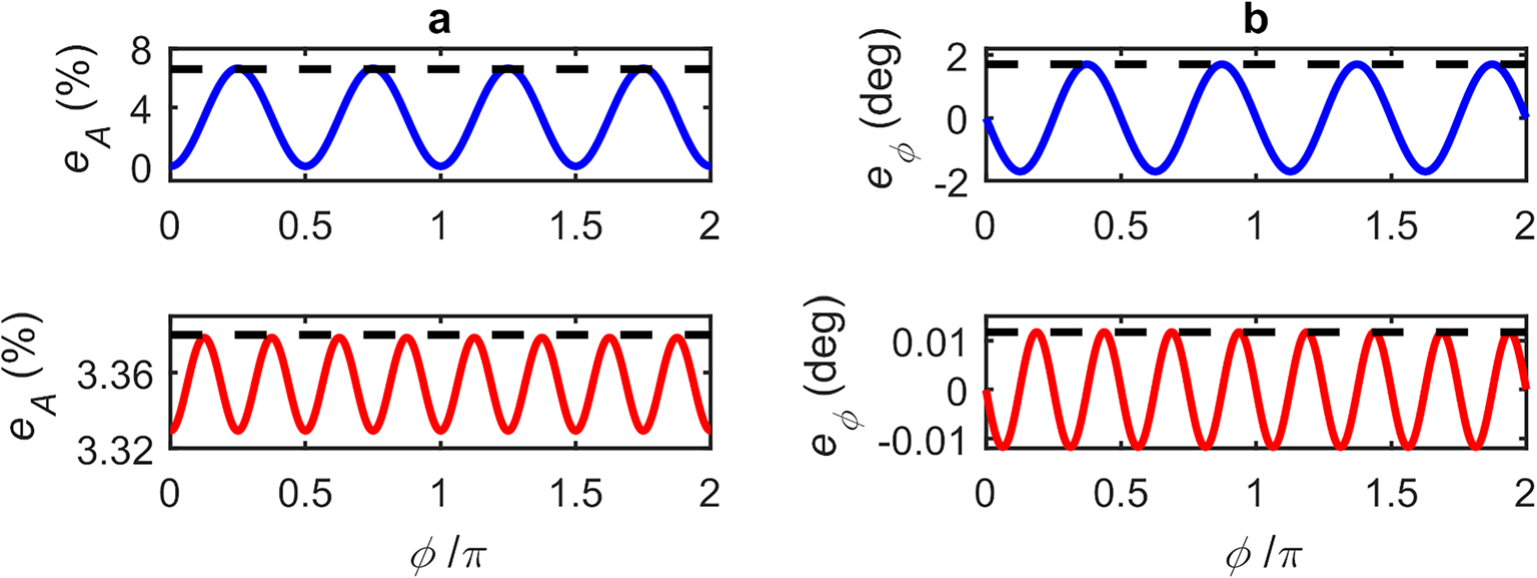
(**a**) Variation of the amplitude error as a function of the phase response of the target, $\phi$, for exemplar cases in which ${\varvec{N}} = 4$ (top panel) and ${\varvec{N}} = 8$ (bottom panel). The corresponding maximum amplitude errors are ${\varvec{e}}_{{{\varvec{A}},{\mathbf{max}}}} =$6.6% and 3.38%, respectively, as shown by the horizontal dashed lines. (**b**) Variation of the phase error for the same ${\varvec{N}}$ as (**a**). The corresponding maximum phase errors are ${\varvec{e}}_{{\phi ,{\mathbf{max}}}} =$1.7° and 0.011°, respectively, as shown by the horizontal dashed lines.

**Figure 4:**
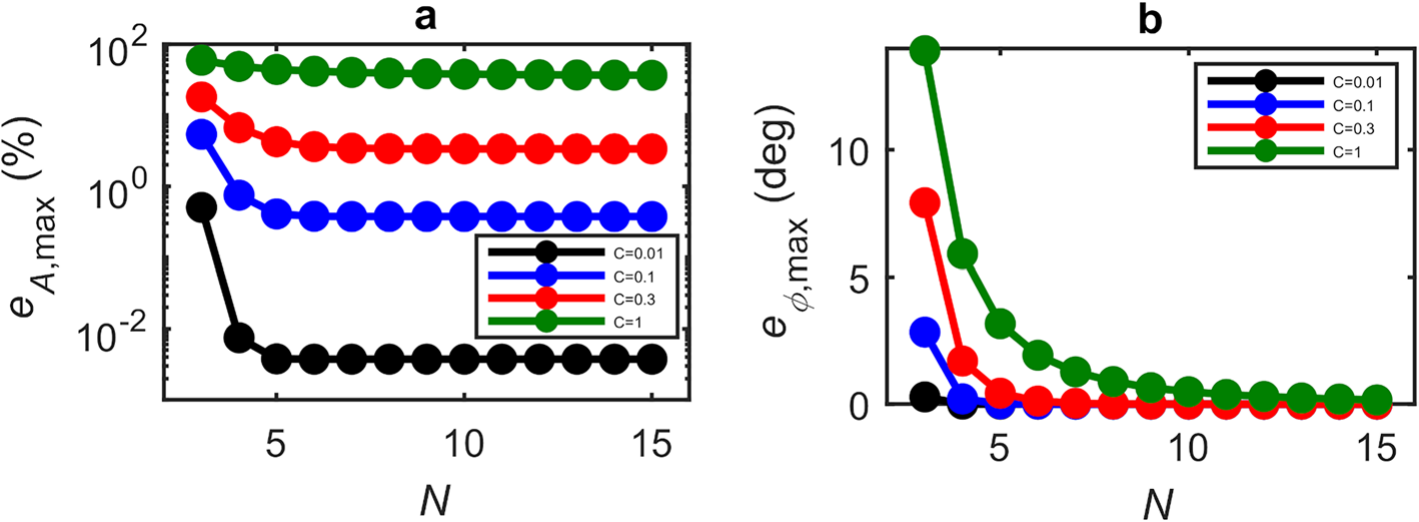
(**a**) Variation of the maximum amplitude error with the number of measurement points ${\varvec{N}}$, for feedback parameters ${\varvec{C}} =$0.01 (black circles), ${\varvec{C}} =$0.1 (blue circles), ${\varvec{C}} =$0.3 (red circles) and ${\varvec{C}} =$1 (green circles). (**b**) Variation of the maximum phase error for the same values of ${\varvec{C}}$. The solid lines are intended only to aid visualisation.

A major benefit of our approach for determining magnitude and phase parameters in LFI is that, under weak levels of feedback, the GPSA remains robust even for small values of $N$. Outside the limit of large $N$, however, the magnitude and phase errors are also dependent on the phase response of the target $\phi$. Equivalently this dependence can be viewed as originating from the choice of phase points $\phi_{i}$ (and hence the choice of $\left( {\phi_{L,s} - { }\phi { }} \right)$) at which the SM voltage signal is sampled, which becomes more critical as the signal departs further from a cosinusoidal dependence on $\phi_{L,s}$. This effect is illustrated in Fig. 2, which shows an exemplar synthesised SM voltage signal along with three possible sets of $\phi_{i}$, each with differing values of $\phi_{i = 0}$, for the case $N =$4. Also shown are the corresponding functions $V_{{{\text{SM}},{\text{m}}}} = \beta_{m} \cos \left( {\phi_{L,s} - \phi_{m} } \right)$ determined by applying the GPSA to each of these sets, revealing the variation in the extracted values of $\beta_{m}$ and $\phi_{m}$. This variation in $\beta_{m}$ and $\phi_{m}$ is further illustrated in Fig. 3a and b, which show typical examples of how the error values $e_{A}$ and $e_{\phi }$ vary with the phase response of the target $\phi$, for the case in which $\phi_{i}$ is arbitrarily fixed with $\phi_{i = 0} = 2\pi m$. Both $\beta_{m}$ and $\phi_{m}$ (and hence $e_{A}$ and $e_{\phi }$) are seen to vary with a periodicity $2\pi /N$ and with a magnitude that decreases significantly with increasing $N$. The former of these observations is particularly relevant to the typical experimental situation in which the value of $\phi$ (and therefore $\left( {\phi_{L,s} - { }\phi { }} \right)$) is not known. To capture this effect quantitatively we therefore define the *maximum* magnitude error, and *maximum* phase error that can be attained within the range $\phi = 0 \to 2\pi$ as $e_{{A,{\text{max}}}} = \max \left\{ {\left| {e_{A} } \right|} \right\}$ and $e_{{\phi ,{\text{max}}}} = \max \left\{ {\left| {e_{\phi } } \right|} \right\}$, respectively. Figure 4a shows how the value of $e_{{A,{\text{max}}}}$ varies with the number of measurement points $N$, for different levels of feedback and assuming $\alpha =$0. The variation of $e_{{\phi ,{\text{max}}}}$ is similarly shown in Fig. 4b. As expected, in the limit of large $N$ the values of $e_{{A,{\text{max}}}}$ and $e_{{\phi ,{\text{max}}}}$ tend towards those reported in Fig. 1a and b. Crucially, however, it can be seen that for extremely weak feedback ($C \le$ 0.1) small maximum error values are achieved for all values of $N >$ 3; in the case $C =$ 0.1, $e_{{A.{\text{max}}}}$ remains less than 1% and $e_{{\phi ,{\text{max}}}}$ below 1° even down to $N =$4. Moreover, $e_{{\phi ,{\text{max}}}}$ remains below 1° for all $C \le$ 1 with $N =$ 8.

**Figure 5:**
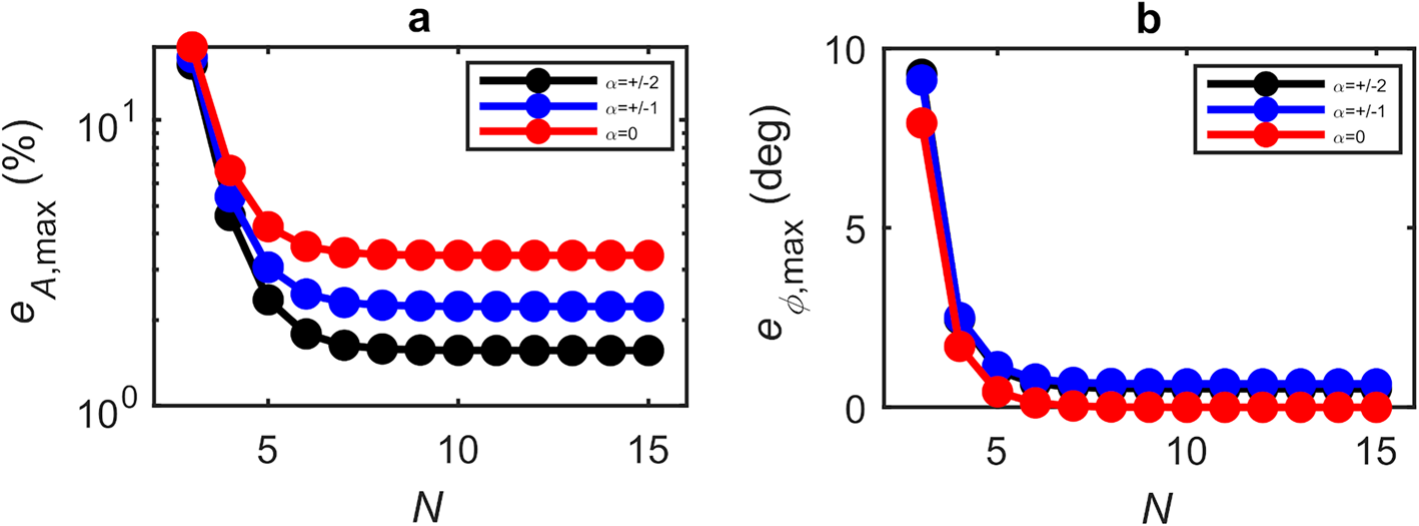
(**a**) Variation of the maximum amplitude error with the number of measurement points ${\varvec{N}}$ for ${\varvec{\alpha}} =$0 (red circles), ${\varvec{\alpha}} = \pm$ 1 (blue circles), ${\varvec{\alpha}} = \pm$2 (black circles). (**b**) Variation of the maximum phase error for the same values of ${\varvec{\alpha}}$. All results are shown for the exemplar case when ${\varvec{C}} =$0.3. The solid lines are intended only to aid visualisation.

Although small values of $\alpha$ in the range ~ − 0.1 to ~ 0.5 are typical for THz QCLs based on a bound-to-continuum active region design^22,44^, significantly larger values have been reported^33,43,45^ for active regions with phonon-assisted electron injection such as that employed in this work. Such values of $\alpha$ are known to impose notable asymmetry on the interferometric fringes observed in LFI, which in turn results in larger values of $e_{{\phi ,{\text{max}}}}$, particularly under stronger feedback. This behaviour is illustrated in Fig. 5b for the exemplar case $C =$0.3. As can be seen, even with an extreme value of $\alpha = \pm$ 2, the maximum phase error remains within ~ 1° of that reported in Fig. 4b for all feedback levels $C \le$ 0.3. At the same time the value of $e_{{A,{\text{max}}}}$ is found to *decrease* as the magnitude of $\alpha$ increases, as shown in Fig. 5a.

Overall the results presented in Figs. 1, 2, 3, 4 and 5 confirm that the GPSA is well suited for reliably extracting both magnitude and phase parameters from typical SM voltage signals acquired over a range of feedback levels and linewidth enhancement factors. Moreover, our approach remains robust even in the limit of small $N$. Notably this is particularly true in the case of extremely weak feedback, as may typically be encountered in THz-s-SNOM where the scattering efficiency of the tip is extremely low. In such cases, where commonly $C \le$ 0.1^33,41,43^, our analysis predicts that magnitude and phase errors far smaller than 1% and 1°, respectively, may be attainable with $N =$4 (see Figs. 4 and 5). It should be noted, however, that the errors reported here represent the fundamental limits of the GPSA in its analysis of LFI fringes under the assumption of an idealised SM response. Larger errors can be expected experimentally due to uncertainty in measurement of the laser response arising, for example, from laser voltage noise. Additional sources of error may also arise from laser frequency noise, occurring for example through thermal drift of the laser operating temperature, which will impact control of the round-trip phase $\phi_{L}$. Nevertheless, as will be shown below, our technique remains robust in experimental situations even with small $N$.

### Far-field LFI using a GPSA

The applicability of the use of the GPSA for determination of magnitude and phase parameters in LFI was investigated initially using a far-field optical feedback system employing a QCL emitting at 3.52 THz (see Methods). The THz beam was focused onto a plane mirror in the far-field of the laser, aligned so that the reflected radiation was reinjected into the laser cavity. Optical feedback to the laser was modulated at a frequency 1 kHz using an optical chopper positioned in the external cavity between laser facet and mirror. The SM signal was recorded by lock-in detection of the QCL terminal voltage referenced to the chopper frequency. Interferometric data was acquired using an all-electronic method of LFI that exploits the tunability of the QCL emission frequency with current^46^. In this approach, the QCL frequency is tuned by a sequential stepping of the QCL driving current over $N$ equally spaced values according to Eq. 14, with the demodulated SM voltage being recorded at each current step.

**Figure 6:**
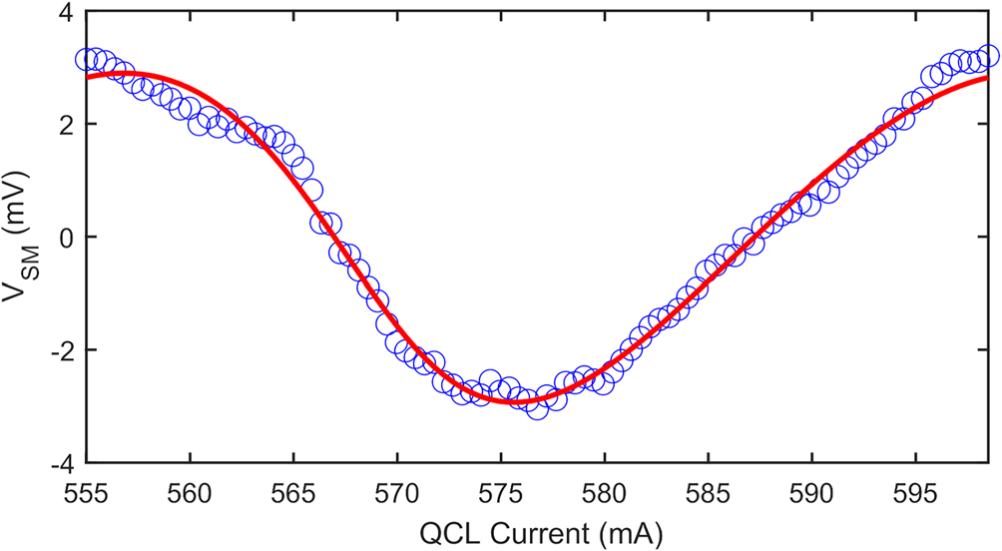
Self-mixing voltage measured as a function of laser driving current, showing one exemplar interferometric fringe obtained by far-field LFI (blue circles). Also shown is a fit to the L–K model (Eq. 1), in which ${\varvec{\beta}} =$ 2.91 mV and $\phi =$− 2.8°.

Figure 6 shows an exemplar single fringe acquired with 94 measurement points. Also shown is a fit to the L–K model (Eq. 1) from which the feedback parameter $C=$ 0.24 and linewidth enhancement factor $\alpha \hspace{0.17em}=\hspace{0.17em}$1.9 are determined. This fit also yields a magnitude $\beta =$ 2.91 mV and phase $\phi \hspace{0.17em}=\hspace{0.17em}$− 2.8, which are regarded as estimates of the true values to which the results of the GPSA analysis can be compared.

**Figure 7:**
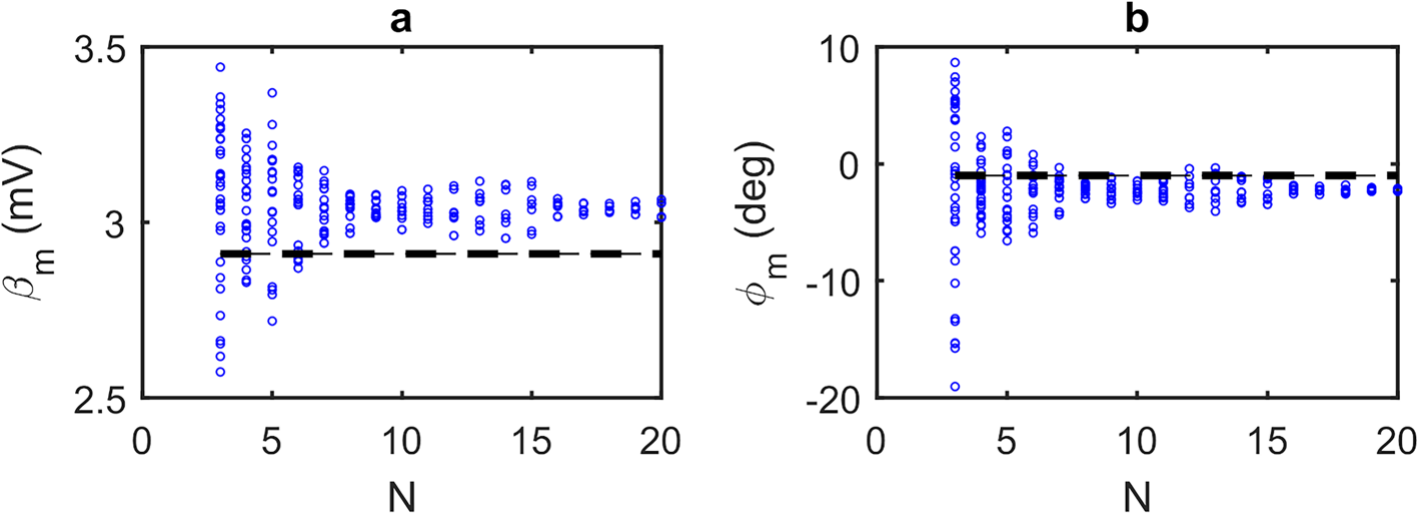
(**a**) Magnitude ${\varvec{\beta}}_{{\varvec{m}}}$ and (**b**) phase $\phi_{{\varvec{m}}}$ values determined by applying the GPSA to the data in Fig. 6, for ${\varvec{N}}$ in the range 3–20. For each value of ${\varvec{N}}$ there exists multiple possible subsets of phase sampling points corresponding to differing positions of the ${\varvec{N}}$ points along the fringe, each of which yield a different pair ${\varvec{\beta}}_{{\varvec{m}}}$ and $\phi_{{\varvec{m}}}$ values. Also shown (dotted lines) are the ‘true’ values of magnitude and phase as determined from the fit shown in Fig. 6.

Using the data presented in Fig. 6 we can extract multiple sets of discrete SM voltage measurements, ${V}_{{\text{SM}},i}$ with $i=0\to (N-1)$, each with progressively reducing value of $N$. Similarly to the situation depicted in Fig. 2, within each set there furthermore exists multiple possible subsets of the $N$ phase sampling points ${\phi }_{i}$, each with differing values of ${\phi }_{i=0}$ i.e. with differing positions of the $N$ points along the fringe. It should be noted these subsets are conceptionally equivalent to sets of measurements acquired with a fixed value ${\phi }_{i=0}$ but varying values of $\left({\phi }_{i}- \phi \right)$ which may arise due to variation in the phase response of the target $\phi$. These subsets thereby reproduce the typical experimental situation in which $\phi$ is not known in advance. For each subset generated in this way, magnitude ${\beta }_{m}$ and phase ${\phi }_{m}$ values were determined using the GPSA with values of $N$ in the range 3–20 (see Methods). Figure 7 shows the results of this analysis. Also highlighted are what are regarded as the ‘true’ values of the magnitude and phase, $\beta$ and $\phi$ respectively, as determined from the fit to the L–K model shown in Fig. 6.

**Figure 8:**
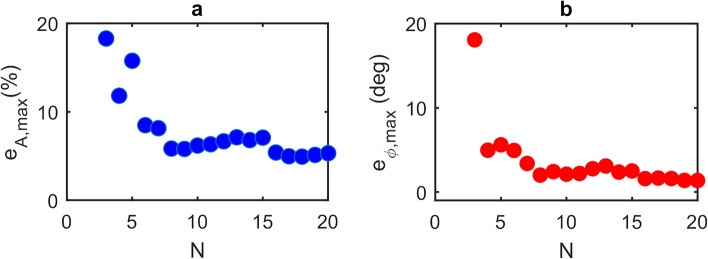
(**a**) Maximum magnitude error ${\varvec{e}}_{{{\varvec{A}},{\mathbf{max}}}} = \max \left\{ {\left| {\left( {\frac{{{\varvec{\beta}}_{{\varvec{m}}} - {\varvec{\beta}}}}{{\varvec{\beta}}}} \right)} \right|} \right\}$ calculated from the magnitude values ${\varvec{\beta}}_{{\varvec{m}}}$ determined from the GPSA (shown in Fig. 7) and expressed as a percentage error relative to the magnitude ${\varvec{\beta}}$ determined from the fit shown in Fig. 6; (**b**) maximum phase error ${\varvec{e}}_{{\phi ,{\mathbf{max}}}} = \max \left\{ {\left| {\phi_{{\varvec{m}}} - \phi } \right|} \right\}$ calculated from the phase values $\phi_{{\varvec{m}}}$ determined from the GPSA (shown in Fig. 7) and the phase $\phi$ determined from the fit shown in Fig. 6.

As can be seen, and in agreement with the analysis presented in Figs. 2 and 3, each subset of measurements ${V}_{{\text{SM}},i}$ yield different values of magnitude ${\beta }_{m}$ and phase ${\phi }_{m}$, the range of which varies with $N$. Furthermore, as $N$ increases this range converges on values close to the true values $\beta$ and $\phi$. Also evident in Fig. 7 and as elucidated by Fig. 1, is that the values determined from the GPSA in the limit of large $N$ deviate slightly from $\beta$ and $\phi$ due to the non-cosinusoidal nature of the fringes. This behaviour is summarised in Fig. 8 which display the maximum magnitude error and maximum phase error, respectively, for given values of $N$. Here ${e}_{A,{\text{max}}}$ and ${e}_{\phi ,{\text{max}}}$ have been estimated from the range of values ${\beta }_{m}$ and ${\phi }_{m}$ shown in Fig. 7, and can be used as an indication of the accuracy of the GPSA when applied to the experimental LFI signals. For large $N>$ 15 these errors found experimentally converge on values of ${e}_{A,{\text{max}}}\approx$ 5% and ${e}_{\phi ,{\text{max}}}\approx$ 1.6° , which are slightly larger than the values ${e}_{A,{\text{max}}}=$ 1% and ${e}_{\phi ,{\text{max}}}=$ − 0.4° predicted for synthesised LFI signals with the same $C$ and $\alpha$ (see Fig. 1). This discrepancy can be explained due to the presence of voltage and frequency noise in the experimental LFI signals, which arise primarily from laser driver current noise and thermal instability of the QCL. The experimental LFI signals are furthermore susceptible to small variations in the current tuning coefficient $\gamma$ across the range of laser driving currents. Such effects may cause deviations of ${V}_{{\text{SM}},i}$ from that predicted by the L–K model under the assumption of constant $\gamma$, as seen for example in Fig. 6 at driving currents ${I}_{i}\approx$~560 mA. This will adversely influence the values of ${\beta }_{m}$ and ${\phi }_{m}$ obtained from the GPSA for certain combinations of ${\phi }_{i=0}$ and $N$. In turn this will manifest as increased values of ${e}_{A,{\text{max}}}$ and ${e}_{\phi ,{\text{max}}}$, the degree of which will also vary with $N$. This phenomenon may be responsible for the apparent enhanced dependency of ${e}_{A,{\text{max}}}$ on $N$ observed in Fig. 8a, when compared to that predicted in Fig. 4a. Nevertheless, as can be seen, even with $N=$ 4 the experimental errors remain low (${e}_{A,{\text{max}}}<$ 12% and ${e}_{\phi ,{\text{max}}}<$ 5°), which may be considered suitable for many experimental situations. Ultimately the choice of $N$ adopted experimentally will be a compromise between the required accuracy and data acquisition time.

### THz-s-SNOM using a GPSA

**Figure 9:**
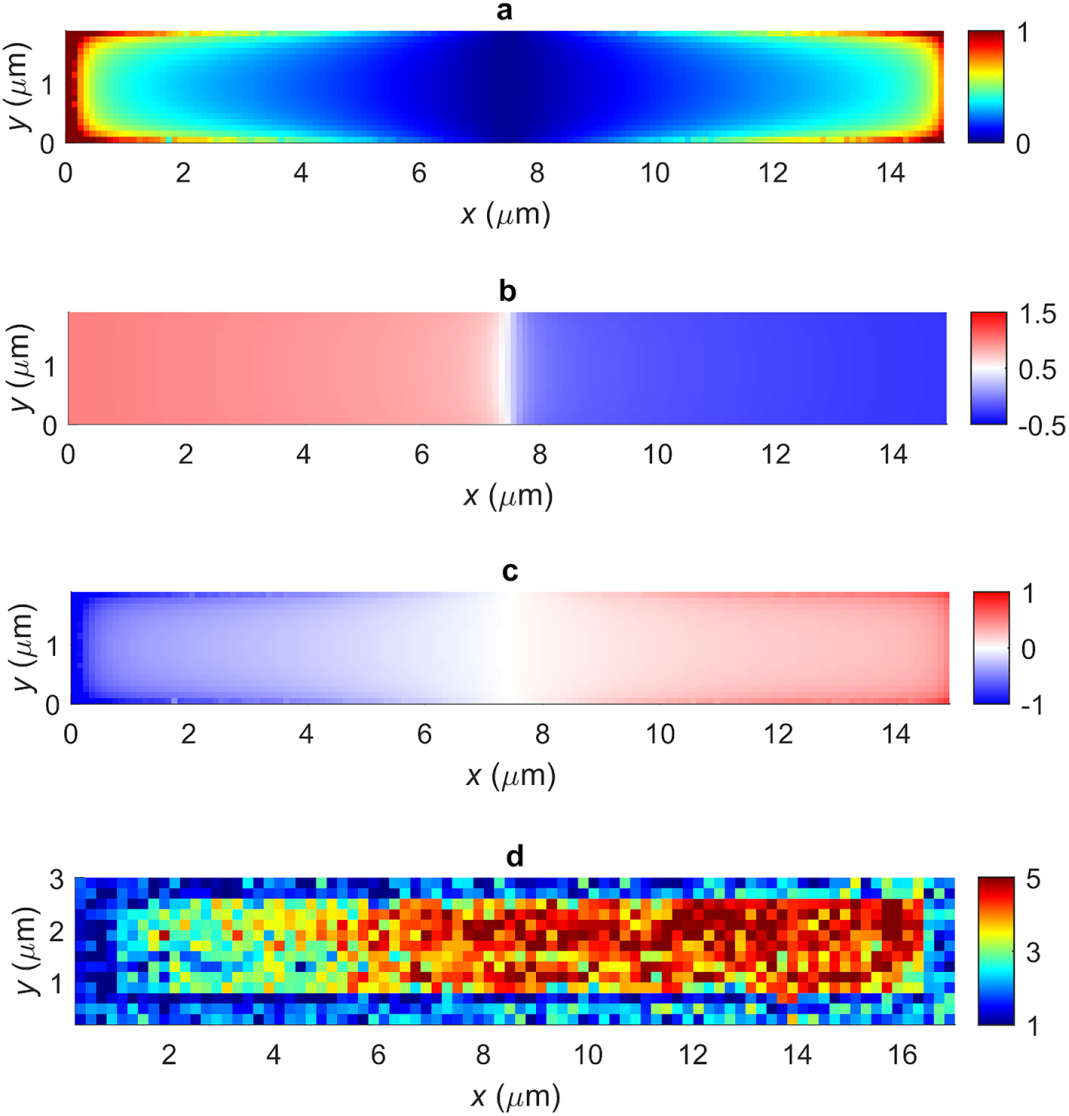
Finite element simulations showing the spatial variation of the (**a**) magnitude ${\varvec{\beta}}_{{\varvec{z}}}$ (a.u.), (**b**) phase $\phi_{{\varvec{z}}}$ (rads/${\varvec{\pi}}$) and (**c**) real part ${\varvec{Re}}\left( {{\varvec{\beta}}_{{\varvec{z}}} {\varvec{e}}^{{ - {\varvec{i}}\phi_{{\varvec{z}}} }} } \right)$ (a.u.) of the out-of-plane field in the *x*–*y* plane 20 nm above the DA, when illuminated under oblique incidence at a frequency 3.45 THz. (**d**) Terahertz image of the dipole antenna structure obtained by THz-s-SNOM, in which the antenna is obliquely illuminated with p-polarised radiation at a frequency 3.45 THz. The colour scale represents the self-mixing voltage derived from the ${\varvec{n}} =$ 2 harmonic of the laser voltage, measured at a single laser driving current. The signal comprises of components capturing both the near-field dipole interaction between tip and sample surface, as well as the spatial distribution of the out-of-plane field supported by the sample under resonant excitation.

To demonstrate coherent near-field imaging using LFI in conjunction with the GPSA, a target consisting of a simple dipole antenna (DA) structure was chosen. This structure comprised a 15 μm $\times \hspace{0.17em}$2 µm gold-on-silicon antenna engineered to support a plasmonic resonance at the frequency 3.45 THz (see Methods). Figure 9a shows a spatial map of the out-of-plane field ${E}_{z}$ measured 20 nm above the sample surface, obtained from finite-element method (FEM) simulations of the DA structure when illuminated obliquely by a p-polarized excitation beam with an in-plane field component oriented along the long axis of the antenna. Also shown in Figs. 9b and c are the corresponding magnitude and phase of the field. The field is strongly enhanced at both ends of the structure, with a π radian phase difference between the two ends, which is characteristic of a dipolar plasmonic mode being excited in the structure.

Near-field images of the DA structure were obtained using a THz-s-SNOM system described elsewhere^32^. Briefly, radiation emitted from a 3.45 THz QCL was collected and focused to the ~ 20 nm apex of the near-field microscope probe, which was positioned in the near-field of the sample surface. Radiation scattered to the far-field by the probe was coupled back to the QCL along the same optical path as the incident beam and reinjected to the laser cavity. To isolate the signal component arising from the near-field interaction between the probe and sample, the microscope probe was operated in tapping mode and the QCL terminal voltage was demodulated at harmonics of the tip tapping frequency. By raster-scanning the sample in two dimensions, images with deeply sub-wavelength resolution could thereby be obtained up to the $n\hspace{0.17em}=\hspace{0.17em}$5 signal harmonic. Figure 9d shows an exemplar image of the DA obtained from the $n\hspace{0.17em}=\hspace{0.17em}$2 signal and with the QCL operated at a single constant driving current. A clear signal contrast between opposing ends of the DA can be observed due to the excitation of a resonant mode in the structure, in agreement with the theoretical predictions (see Fig. 9). Also evident in this image is a non-negligible signal component arising from the near-field dipole interaction between the tip and the dielectric sample (see below).

**Figure 10:**
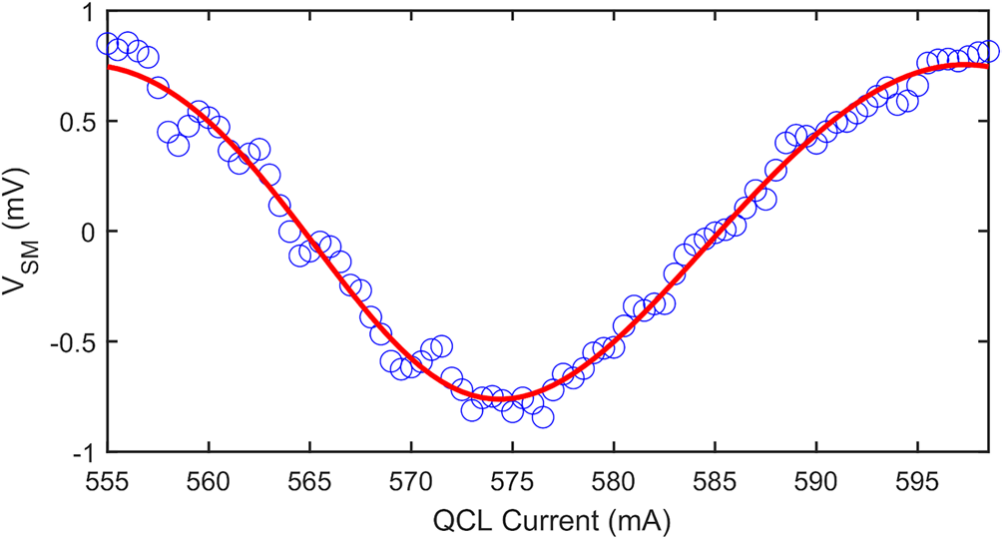
Self-mixing voltage obtained from demodulation of the laser voltage at the ${\varvec{n}} =$ 3 harmonic of the tip tapping frequency, measured as a function of laser driving current (blue circles). Also shown is a fit to the L–K model (Eq. 1), in which ${\varvec{\beta}} =$ 0.76 mV and $\phi =$ 14.7°.

In order to resolve both the magnitude $${\beta }_{m}$$ and phase $${\phi }_{m}$$ of the field scattered from the s-SNOM probe an interferometric fringe can be generated at the chosen sampling position by stepping the laser driving current according to Eq. 13, with $${V}_{{\text{SM}},i}$$ being recorded at each current. Figure 10 shows an exemplar single fringe acquired in this way with 87 measurement points. Also shown is a fit to the L–K model (Eq. 1) from which the parameters $$ C =$$0.13 and $$ \alpha = $$ 0.95 are determined.

**Figure 11:**
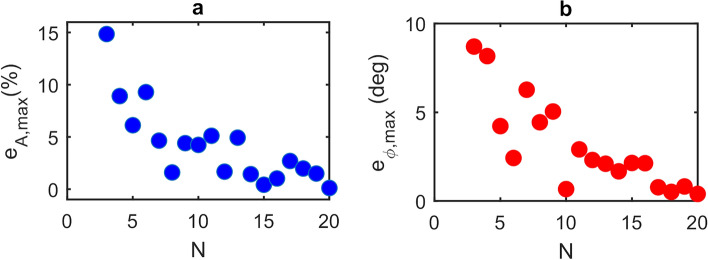
(**a**) Maximum magnitude error ${\varvec{e}}_{{{\varvec{A}},{\mathbf{max}}}}$ and (b) maximum phase error ${\varvec{e}}_{{\phi ,{\mathbf{max}}}}$ determined from the magnitude and phase values obtained from the GPSA when applied to the data shown in Fig. 10, expressed relative to those determined from the fit shown in Fig. 10.

Following the same procedure as described previously the data in Fig. 10 can be used to estimate the maximum magnitude error and maximum phase error when applying the GPSA to these experimental s-SNOM signals. Figure 11 shows the results of this analysis for values of $$ 3 \le N \le 20$$. The errors associated with both of these quantities follow similar trends to those observed in the case of far-field LFI signals (Fig. 8), with $$e_{{A,{\text{max}}}}$$ and $$e_{{\phi ,{\text{max}}}}$$ converging on values < 1% and < 1°, respectively, for large $$N >$$ 16. However, despite the smaller value of $$C$$ in the near-field case, larger errors are generally observed compared to those reported for the far-field case. This is primarily due to the significantly lower signal-to-noise ratio common to THz-s-SNOM measurements, which arises from the weak scattering efficiency of the tip as well as signal demodulation at higher harmonics of the tip modulation frequency. Nevertheless, even for small $$N = $$4, moderate error values $$e_{{A,{\text{max}}}} \approx 9{\text{\% }}$$ and $$e_{{\phi ,{\text{max}}}} \approx 8$$° are attained, which are sufficiently low to enable reliable magnitude and phase extraction of near-field LFI signals using the GPSA.

To demonstrate coherent near-field imaging, a set of interferometric fringes with chosen $$N$$ were acquired at each pixel during a single raster-scan of the sample. By applying the GPSA to the interferometric data obtained at each pixel, two-dimensional maps of both magnitude $$\beta_{m}$$ and phase $$\phi_{m}$$ of the scattered field were thereby obtained. It has been shown previously^32^ that the self-mixing voltage signal in THz-s-SNOM comprises two signal components according to Eq. 15. The first of these is principally excited by p-polarised components of the incident THz field and captures information about the local permittivity of the sample. In contrast the second component is insensitive to the bulk material properties but captures the spatial distribution of both the magnitude $$\beta_{z}$$ and phase $$\phi_{z}$$ of the out-of-plane field $$E_{z} $$ supported by the sample due to resonant excitation by in-plane components of the incident field. By exploiting its spatial symmetry the former of these can be removed from the total measured signal (see Methods), thereby isolating the complex amplitude $$\beta_{z} e^{{ - i\phi_{z} }}$$ associated with the out-of-plane field for each pixel.

**Figure 12:**
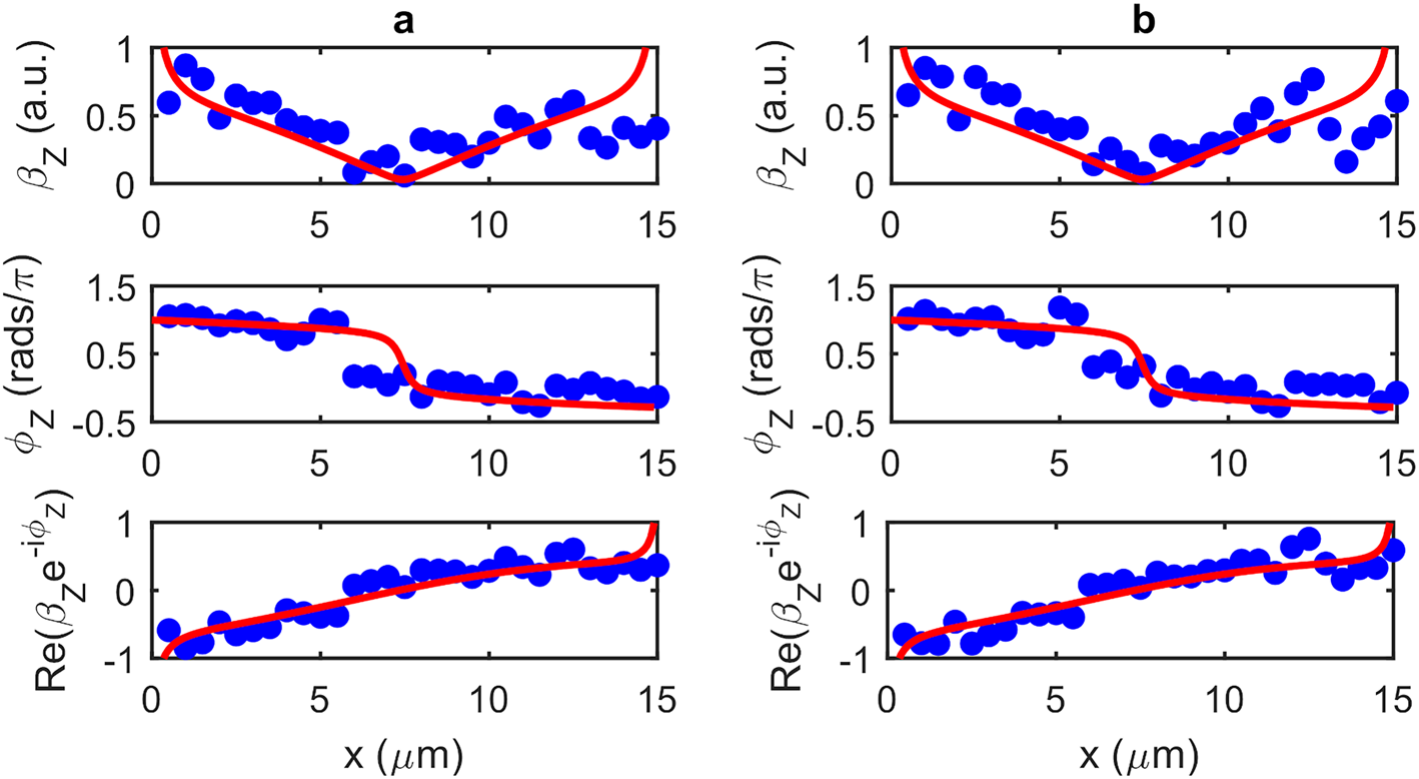
Magnitude ${\varvec{\beta}}_{{\varvec{z}}}$, phase $\phi_{{\varvec{z}}}$ and real part ${\varvec{Re}}\left( {{\varvec{\beta}}_{{\varvec{z}}} {\varvec{e}}^{{ - {\varvec{i}}\phi_{{\varvec{z}}} }} } \right)$ of the out-of-plane field component associated with the plasmonic dipole mode excited in the DA under resonant excitation by THz radiation. Blue circles show measured values, obtained by THz-s-SNOM and applying the GPSA with (**a**) ${\varvec{N}} =$ 15 and (**b**) ${\varvec{N}} =$ 4 measurements per pixel, plotted as a function of position along the principal axis of the antenna. Also shown (red lines) are the corresponding values derived from FEM simulations shown in Fig. 9.

**Figure 13:**
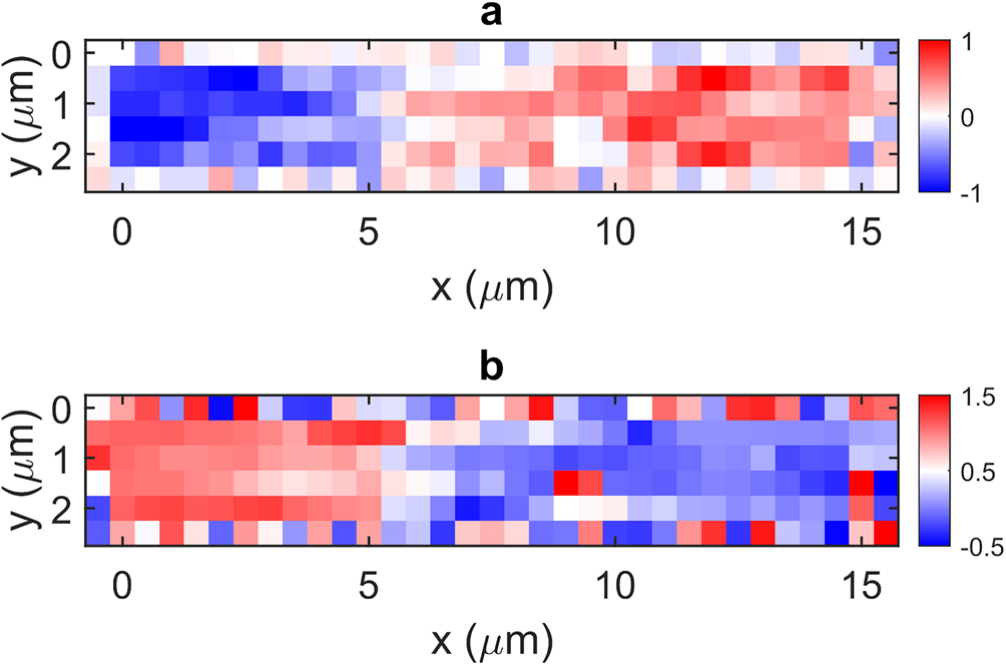
Two-dimensional images showing the (**a**) real part ${\varvec{Re}}\left( {{\varvec{\beta}}_{{\varvec{z}}} {\varvec{e}}^{{ - {\varvec{i}}\phi_{{\varvec{z}}} }} } \right)$ (a.u.) and (**b**) phase $\phi_{{\varvec{z}}}$ (rads/${\varvec{\pi}}$) of the out-of-plane field component supported by the DA, obtained by THz-s-SNOM and applying the GPSA with ${\varvec{N}} =$4 measurements per pixel. The first and last pixels of each row and column correspond to the substrate region of the sample. Both images have been generated by concatenating 1D scans taken at different y-positions on the sample.

One-dimensional coherent measurements of the DA were performed initially by scanning the structure parallel to its principal-axis, wherein at each pixel a set of $N =$15 data points were acquired spanning one interferometric fringe. Figure 12a shows the magnitude, phase and real part of the complex amplitude $s_{z} e^{{ - i\varphi_{z} }}$ obtained using the procedure described above. For comparison, an equivalent scan with $N =$4 is shown in Fig. 12b. Also plotted in these Figures are the corresponding values of the out-of-plane electric field component $E_{z}$ associated with the plasmonic mode calculated from FEM simulations (see Fig. 9). In calculating the simulated phase it is necessary to also account for the phase retardation arising from the oblique illumination geometry, which causes the phase of the excitation field to vary as the spatially-structured sample is scanned within the beam. In our experimental geometry this phase retardation $\Delta \phi$ is described by

5 $$ {\Delta }\phi = - \frac{2\pi }{\lambda }x\sin \theta $$

where $x$ is the coordinate along the principal axis of the DA, $\theta$ is the incident angle of the THz beam and $\lambda$ is its wavelength. The varying of this positional-dependent phase also contributes to the image contrast seen in Fig. 11. Overall it can be seen that the experimental measurements show good agreement with theoretical expectations. The drop in signal magnitude observed in both Figs. 12a and b in the region $x \ge$12.5 μm is ascribed to shadowing of the sample by the s-SNOM probe, which causes variation in the incident beam intensity as the sample is scanned. This effect will also cause an underestimation of the signal contribution arising from the near-field dipole interaction between illuminated tip and sample surface (i.e. the term $\beta_{\varepsilon } e^{{ - i\phi_{\varepsilon } }}$ in Eq. 16), which we obtain from the spatial average of the signal recorded across all gold regions of the sample. This in turn may explain the slight discrepancy between the spatial position of the $\pi$ to 0 phase step observed in simulations and experiment; we note that determination of the phase $\phi_{\varepsilon }$ is particularly sensitive to the value of $\beta_{\varepsilon } e^{{ - i\phi_{\varepsilon } }}$ in the central region of the sample where the magnitude $\beta_{z}$ is small. As a final observation, it can be seen in Fig. 12 that there is little to no discrepancy between the $N =$15 and $N =$4 scans. It can therefore be concluded that $N =$ 4 phase measurement points is sufficient for this technique to capture magnitude and phase of the out-of-plane field. By repeating this measurement procedure across several adjacent rows of pixels, a two-dimensional coherent image of the DA was also acquired as shown in Fig. 13. These images reveal a clear signal contrast between opposite ends of the DA, which is characteristic of the dipolar plasmonic resonance excited in the structure in concurrence with the simulations shown in Fig. 9.

## Conclusion

In summary, we have demonstrated the use of a generalised phase-stepping algorithm to extract magnitude and phase information from interferometric fringes acquired by LFI with only a small number of sampling points. The applicability of this approach has been investigated theoretically for different levels of optical feedback, different laser parameters, and for different sampling conditions. Our analysis reveals how the accuracy of this approach reduces for decreasing sampling points $N$, as well as increasing feedback strength due to the associated asymmetry induced in LFI signals. We have determined that both magnitude and phase values can be measured with sufficient accuracy over a wide range of weak feedback levels and linewidth enhancement factors typical to common experimental situations, even down to $N =$4.

Our approach based on the GPSA has been validated experimentally, initially through the analysis of SM voltage signals measured from a THz-frequency QCL in a far-field LFI geometry. We have thereby demonstrated that for an exemplar value of feedback parameter $$C =$$0.24, the magnitude of phase can be determined experimentally with an inaccuracy of only < 12% and < 5°, respectively, with only $$N = $$4 measurement points, but decreasing to < 5% and < 2° for larger $$N$$. We have furthermore investigated the applicability of our approach for coherent THz-s-SNOM employing an all-electronic method of interferometric fringe generation that exploits frequency tuning of the QCL. Using this technique we have successfully demonstrated deeply sub-wavelength-resolution coherent imaging of the out-of-plane field supported by a THz micro-resonator under resonant excitation. A comparison between images acquired using different $$N$$ confirms that $$N = $$4 measurements per pixel is sufficient to extract magnitude and phase information, with little impact on the image quality.

Our new approach enables significantly reduced optical sampling and acquisition times in LFI, whilst also avoiding the need for fitting to complex models of lasers under optical feedback in post-processing. One notable application that will benefit from reduced sampling bandwidths is fast THz imaging^35^, which demands the capture and electronic processing of high-bandwidth LFI signals in real-time, thereby limiting attainable imaging rates. We also anticipate significant benefits for coherent THz-s-SNOM, in which imaging rates have previously been severely limited owing to the need for long averaging times and acquisition of a large number of measurements per imaging pixel. In comparison to previous implementations, for example^32^ which adopted 106 measurements per pixel, our technique permits a proportionate decrease in total imaging time, which scales with the total number of measurements. Addressing this bottleneck will open up new opportunities for THz measurements on the micro- and nano-scale. More widely we envisage our approach will open up opportunities for fast coherent sensing, not only at THz frequencies but across the visible and infra-red spectrum.

## Methods

### Numerical synthesis of laser feedback interferograms

The response of a laser subject to optical feedback can be described using the well-established rate equation model for the complex field and carrier density proposed by Lang and Kobayashi, which includes the influence of feedback through a time-delayed field term ^42^. Under the steady-state condition these rate equations reduce to a set of equations for the laser frequency $\nu$ and the threshold carrier density $n$^1,2^:

6 $$ 2\pi \tau_{{{\text{ext}}}} \left( {\nu_{s} - \nu } \right) = C\sin \left( {2\pi \nu \tau_{{{\text{ext}}}} + \arctan \left( \alpha \right)} \right), $$

7 $$ n - n_{s} = - \tilde{\beta }\cos \left( {2\pi \nu \tau_{{{\text{ext}}}} } \right) $$

where the subscript *s* indicates values for the solitary laser without feedback, $\tilde{\beta }$ represents the coupling rate of feedback relative to the rate of carrier density gain, $\alpha$ is the linewidth enhancement factor, and $\tau_{{{\text{ext}}}}$ is the round-trip delay in the external cavity given by $\tau_{{{\text{ext}}}} = 2L_{{{\text{ext}}}} /c$. The dimensionless feedback parameter $C$ defines the strength of optical feedback, and is proportional to the reinjected field that couples coherently to the laser mode after scattering from the probe.

The change in laser voltage under feedback can be assumed to be proportional to the change in carrier density for small perturbations^3^. Following Eq. 7 it can thereby be seen that the SM voltage signal in the coherent s-SNOM implementation can be expressed according to Eq. 1, with $\nu$ obtained from the solution to Eq. 6. Laser feedback interferograms are simulated numerically in this way, for chosen values of $C$ and $\alpha$, over a range of phase values $\phi_{L,s} = \frac{{4\pi L_{{{\text{ext}}}} \nu_{s} }}{c} = 2\pi m \to 2\pi \left( {m + 1} \right)$ where $m$ is an integer.

### Generalised phase-stepping algorithm

The generalized phase-stepping algorithm used in this work^36^ models the self-mixing voltage signal as a series of $N$ discrete voltages ${\text{V}}_{{{\text{SM}},i}}$ measured at phase points $\phi_{i}$, where $i = 0, 1 \ldots N - 1$, according to Eq. 2.

To solve for $$ a_{0}$$, $$a_{1}$$ and $$ a_{2}$$ according to the least-squares method we use the matrix equation

8 $$ {\varvec{A}}\left( {\phi_{{\varvec{i}}} } \right){\varvec{a}} = {\varvec{b}}\left( {\phi_{{\varvec{i}}} } \right) $$

where

9 $$ {\varvec{A}}\left( {\phi_{{\varvec{i}}} } \right) = \left[ {\begin{array}{*{20}c} N & {\mathop \sum \limits_{i = 0}^{N - 1} \cos \phi_{i} } & {\mathop \sum \limits_{i = 0}^{N - 1} \sin \phi_{i} } \\ {\mathop \sum \limits_{i = 0}^{N - 1} \cos \phi_{i} } & {\mathop \sum \limits_{i = 0}^{N - 1} \cos^{2} \phi_{i} } & {\mathop \sum \limits_{i = 0}^{N - 1} \cos \phi_{i} \sin \phi_{k} } \\ {\mathop \sum \limits_{i = 0}^{N - 1} \sin \phi_{i} } & {\mathop \sum \limits_{i = 0}^{N - 1} \cos \phi_{i} \sin \phi_{i} } & {\mathop \sum \limits_{i = 0}^{N - 1} \sin^{2} \phi_{i} } \\ \end{array} } \right], $$

10 $$ {\varvec{a}} = \left( {\begin{array}{*{20}c} {a_{0} } \\ {a_{1} } \\ {a_{2} } \\ \end{array} } \right), $$

and

11 $$ {\varvec{b}}\left( {\phi_{{\varvec{i}}} } \right) = \left[ {\begin{array}{*{20}l} {\mathop \sum \limits_{i = 0}^{N - 1} V_{SM, i} } \hfill \\ {\mathop \sum \limits_{i = 0}^{N - 1} V_{SM,i} \cos \phi_{i} } \hfill \\ {\mathop \sum \limits_{i = 0}^{N - 1} V_{SM,i} \sin \phi_{i} } \hfill \\ \end{array} } \right]. $$

If ${\varvec{A}}$ is not ill-conditioned, then we obtain,

12 $$ {\varvec{a}} = \user2{ A}^{ - 1} \left( {\phi_{{\varvec{i}}} } \right){\varvec{b}}\left( {\phi_{{\varvec{i}}} } \right) $$

The magnitude $\beta_{m}$ and phase $\phi_{m}$ of the SM voltage signal can then be obtained from Eqs. 3 and 4, respectively.

### Experimental setup for far-field LFI

The 3.52 THz QCL device used in the far-field system was based on a 10-μm-thick bound-to-continuum active region incorporating a phonon extraction/injection stage. The device was processed into a semi-insulating surface-plasmon ridge waveguide with dimensions 1.8 mm × 155 μm. A grating with periodicity Λ = 11.8 μm was defined in the 150-nm-thick Au layer on top of the ridge, consisting of ~ 1.8-μm-wide regions with no metal and from which the 50-nm-thick n + layer was removed to ensure that the surface plasma cannot be supported.

The QCL was cooled in a continuous-flow helium cryostat and was maintained at a heat sink temperature of 20 K. Radiation from the QCL was collimated and focused using two identical *F*/2 parabolic mirrors onto a plane mirrored target. The beam was reflected back along the same optical path and reinjected into the laser cavity, forming an external cavity of length $L_{{{\text{ext}}}} =$47 cm. The level of optical feedback was controlled using attenuators placed in the beam in the external cavity. To generate one interferometric fringe the QCL driving current was stepped within the range 788–826 mA. Throughout its operation over this range, the QCL maintained lasing on a single longitudinal mode which was tuned by ~ 319 MHz.

### Measurement of laser feedback interferograms

The interferometric signal is obtained from a series of discrete voltage samples $V_{{{\text{SM}},i}}$, where $i = 0 \to \left( {N - 1} \right)$, taken at $N >$ 3 phase points $\phi_{L,s} = \phi_{i}$ equally spaced over a single interferometric fringe. In our case this is achieved through control of the emission frequency of the solitary (unperturbed) QCL, which depends on the laser drive current $I_{i}$ according to the relationship

13 $$ \nu_{s,i} { } = \nu_{s,0} + \gamma \left( {I_{i} - I_{0} } \right), $$

where $$\gamma$$ is the current tuning coefficient and $$\nu_{s,0}$$ is the emission frequency of the solitary laser at a drive current $$ I_{0}$$. It follows from Eqs. 1 and 13 that equally spaced phase points are generated for a series of driving currents given by

14 $$ I_{i} = \frac{i}{N}\frac{2\pi }{{\gamma \tau_{{{\text{ext}}}} }} + I_{0} . $$

In our experiment $I_{0}$ is chosen such that $\nu_{s,0} = \frac{m}{{\tau_{{{\text{ext}}}} }}$, where $m$ is an integer, and $V_{{{\text{SM}}}}$ thereby attains a maximum value for $i =$0 when the phase response of the target $\phi =$0. For s-SNOM measurements this value of $I_{0}$ is determined by measuring a region of the sample where the out-of-plane field is negligible (i.e. for which $\beta_{z} \approx$0).

### Sample fabrication

The dipole antenna resonator structure was fabricated using standard electron-beam lithography on a high resistivity ($> 10000 \;\Omega$ cm) undoped silicon substrate with a thickness 525 ± 25 µm. The thickness of the Ti/Au resonator was ≈ 2 nm/≈ 100 nm, an array of which were patterned across a 2 × 2 mm^2^ region of the substrate with a periodicity of 13 µm.

### Experimental setup for THz-s-SNOM

The 3.45 THz QCL device used for THz-s-SNOM consisted of a 14-μm-thick GaAs/AlGaAs 9-well active region based on LO-phonon-assisted interminiband transitions, which was processed into a semi-insulating surface-plasmon ridge waveguide with dimensions of 1.8 mm × 150 μm. To achieve lasing on a single longitudinal mode a 166-µm-long finite-site photonic lattice was patterned through the top contact layers using focused-ion beam milling^46^. The lattice period was 13.2 µm with a 70% mark-space ratio and a central 8-μm-wide phase defect.

P-polarised radiation from the QCL was focused to the vertically aligned tip of the s-SNOM system at an angle of ~ 54° relative to the surface normal and the length of the external cavity formed between the tip and the QCL facet was L_0_ = 60 cm. The self-mixing signal, arising from the field scattered from the s-SNOM tip and reinjected to the laser cavity, was derived from the QCL terminal voltage which was demodulated at $n =$1–5 harmonics of the tip tapping frequency (Ω ~ 80 kHz) after amplification using an AC-coupled low-noise voltage amplifier.

Single-frequency THz-s-SNOM images of the DA were acquired with a step size of 200 nm, a tip tapping amplitude of ~ 175 nm and an integration time of 200 ms. Coherent THz-s-SNOM images were acquired with the QCL driving current being stepped incrementally in the range 563–605 mA with a current step of 3 mA. A lock-in time constant of 200 ms was used and all $N =$15 data points were acquired over a period of 6000 ms, after which a short delay allowed the s-SNOM probe to move to the location of the subsequent pixel. To generate the $N =$4 data, four equally-spaced data points were extracted from the set of 15 measurements for each pixel, thereby assuming a new current step of 12 mA.

### Generation of out-of-plane field maps

The SM voltage signal recorded at each position on the sample is given in complex notation by^32^

15 $$ V_{SM} = \beta_{m} e^{{i\left( {\phi_{L} - \phi_{m} } \right)}} = \left[ {\beta_{\varepsilon } e^{{ - i\phi_{\varepsilon } }} + \beta_{z} e^{{ - i\phi_{z} }} } \right]e^{{i\phi_{L} }} , $$

from which it follows that

16 $$ \beta_{m} e^{{ - i\phi_{m} }} = \beta_{\varepsilon } e^{{ - i\phi_{\varepsilon } }} + \beta_{z} e^{{ - i\phi_{z} }} $$

Here $\beta_{\varepsilon }$ and $\phi_{\varepsilon }$ are the magnitude and phase, respectively, of the signal contribution arising from the near-field dipole interaction between illuminated tip and sample surface. The parameters $\beta_{z}$ and $\phi_{z}$ are directly related to the magnitude and phase of the out-of-plane field component, $E_{z}$, associated with resonant modes supported by the sample. The field distribution associated with the plasmonic dipole mode excited in the DA exhibits equal magnitude but opposite phase in opposite halves of the structure (i.e. $\beta_{z} e^{{ - i\phi_{z} }}$ is spatially asymmetric) and will therefore spatially average to zero. As such the spatially constant term $\beta_{\varepsilon } e^{{ - i\phi_{\varepsilon } }}$ can be readily estimated from the spatial average of the signal recorded across all gold regions of the sample. Using Eq. 16 this value can then be subtracted from the measured signal $\beta_{m} e^{{ - i\phi_{m} }}$ to isolate the magnitude $\beta_{z}$ and phase $\phi_{z}$ for each pixel.

### Supplementary Information

Supplementary Information.

## Data Availability

The data associated with this paper are openly available from the University of Leeds Data Repository: https://doi.org/10.5518/1432.
