# Supplementary material for: Terahertz microscopy using laser feedback interferometry based on a generalised phase-stepping algorithm
Source: Sci Rep. 2024 Feb 8;14:3274. doi: 10.1038/s41598-024-53448-8 (PMC10853214; doi:10.1038/s41598-024-53448-8)
Supplement: Supplementary file 1 — Supplementary Information. [file 41598_2024_53448_MOESM1_ESM.docx]

**Terahertz microscopy using Laser Feedback Interferometry based on a Generalised Phase-stepping Algorithm**

Daniel Mohun,^1^ Nikollao Sulollari,^1^ Mohammed Salih,^1^ Lianhe H. Li^1^, John E. Cunningham^1^, Edmund H. Linfield,^1^ A. Giles Davies^1^ and Paul Dean^1*^

^1^ School of Electronic and Electrical Engineering, University of Leeds, Leeds, LS2 9JT, UK

*p.dean@leeds.ac.uk

The shape and form of the interferometric fringes acquired by laser feedback interferometry (LFI) are inherently dependent on the feedback parameter $C$ as well as the linewidth enhancement factor of the laser, α, as described by Equation 1. In turn, the laser frequency under optical feedback, $\nu$, is determined by the transcendental excess phase equation (Equation 3), which relates the round-trip phase under feedback $\phi_{L}$ to the phase $\phi_{L,s}=\frac{4\pi L_{\mathrm{ext}}\nu_{s}}{c}$ calculated for the unperturbed frequency of the solitary laser, $\nu_{s}$. Under extremely weak feedback ($C<$0.1) the perturbed laser frequency is approximately equal to that of the solitary laser, $\nu\approx\nu_{s}$, such that $\phi_{L}\approx\phi_{L,s}$. In such cases the self-mixing voltage signal $V_{\mathrm{SM}}$ closely approximates a cosinusoidal dependence on $\phi_{L,s}$, i.e. $V_{\mathrm{SM}}\approx\beta\cos\left( \phi_{L,s}-\phi\right)$.

Figure S1(a) shows $V_{\mathrm{SM}}$ calculated from Equation 1 for the exemplar case in which $C=$0.1 and $\alpha=$0.5, and with amplitude $\beta=$1 and phase $\phi=$0. Also shown is the function $V_{SM,m}=\beta_{m}\cos\left( \phi_{L,s}-\phi_{m} \right)$ in which the magnitude $\beta_{m}=$ 0.997 and phase $\phi_{m}=-$0.057° values have been determined by applying the generalised phase-stepping algorithm (GPSA) to the synthesised LFI signal. In this limit of extremely weak feedback the synthesised LFI signal can be seen to closely resemble a cosinusoidal function, giving rise to small amplitude and phase errors $e_{A}<$ 0.4% and $e_{\phi}<$ 0.06°.

By contrast, Figure S1(b) shows $V_{\mathrm{SM}}$ calculated with $C=$0.5 and $\alpha=$0.5, in which the synthesised LFI signal deviates more noticeably from a cosinusoidal function due to stronger feedback. Nevertheless in this case the GPSA yields the values $\beta_{m}=$ 0.92 and $\phi_{m}=-$1.52°, corresponding to amplitude and phase errors of only $e_{A}<$ 8% and $e_{\phi}<$ 2°.

**Fig. S1 – (a) Synthesised LFI signal with** $\boldsymbol{C=}$ **0.1,** $\boldsymbol{\alpha=}$ **0.5, amplitude** $\boldsymbol{\beta=}$**1 and phase** $\boldsymbol{\phi=}$**0 (black dashed line). Also shown (blue solid line) is the corresponding function** $\boldsymbol{V}_{\mathbf{SM,m}}\boldsymbol{=}\boldsymbol{\beta}_{\boldsymbol{m}}\cos\left( \boldsymbol{\phi}_{\boldsymbol{L,s}}\boldsymbol{-}\boldsymbol{\phi}_{\boldsymbol{m}} \right)$ **plotted using the values of** $\boldsymbol{\beta}_{\boldsymbol{m}}$ **and** $\boldsymbol{\phi}_{\boldsymbol{m}}$ **determined from the GDRA. (b) Synthesised LFI signal with** $\boldsymbol{C=}$ **0.5,** $\boldsymbol{\alpha=}$ **0.5, amplitude** $\boldsymbol{\beta=}$**1 and phase** $\boldsymbol{\phi=}$**0 (black dashed line), as well as the corresponding function** $\boldsymbol{V}_{\mathbf{SM,m}}\boldsymbol{=}\boldsymbol{\beta}_{\boldsymbol{m}}\cos\left( \boldsymbol{\phi}_{\boldsymbol{L,s}}\boldsymbol{-}\boldsymbol{\phi}_{\boldsymbol{m}} \right)$ **determined from the GDRA.**

**
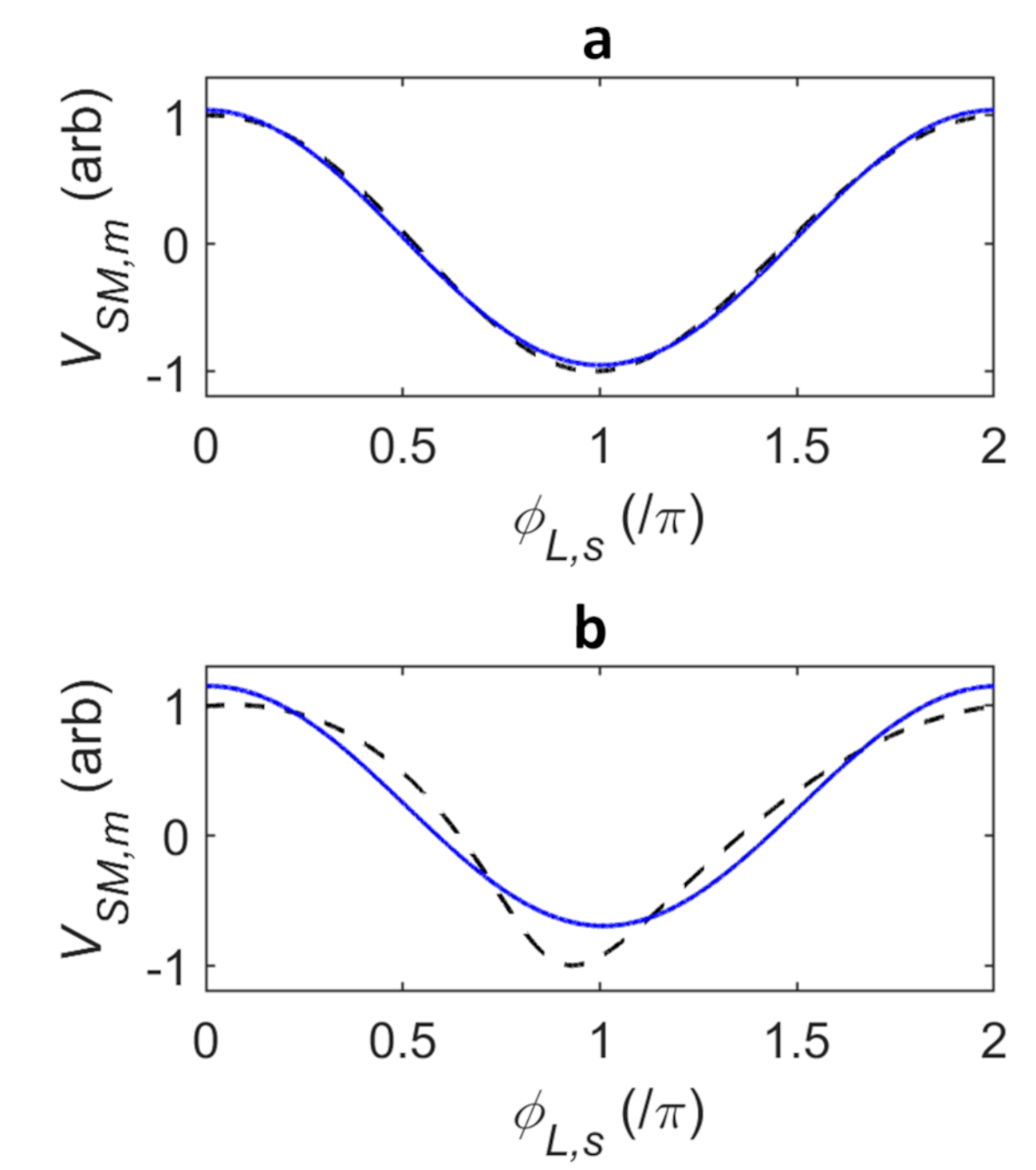
**
